# Invariant Representation Learning in Multimedia Recommendation with Modality Alignment and Model Fusion

**DOI:** 10.3390/e27010056

**Published:** 2025-01-10

**Authors:** Xinghang Hu, Haiteng Zhang

**Affiliations:** 1 School of Materials Science and Engineering, Sichuan University, Chengdu 610065, China; 2017141061004@stu.scu.edu.cn; 2 Academy of Mathematics and Systems Science, Chinese Academy of Sciences, Beijing 100190, China

**Keywords:** multimedia recommendation, model fusion, multimodal representation

## Abstract

Multimedia recommendation systems aim to accurately predict user preferences from multimodal data. However, existing methods may learn a recommendation model from spurious features, i.e., appearing to be related to an outcome but actually having no causal relationship with the outcome, leading to poor generalization ability. While previous approaches have adopted invariant learning to address this issue, they simply concatenate multimodal data without proper alignment, resulting in information loss or redundancy. To overcome these challenges, we propose a framework called M^3^-InvRL, designed to enhance recommendation system performance through common and modality-specific representation learning, invariant learning, and model merging. Specifically, our approach begins by learning modality-specific representations along with a common representation for each modality. To achieve this, we introduce a novel contrastive loss that aligns representations and imposes mutual information constraints to extract modality-specific features, thereby preventing generalization issues within the same representation space. Next, we generate invariant masks based on the identification of heterogeneous environments to learn invariant representations. Finally, we integrate both invariant-specific and shared invariant representations for each modality to train models and fuse them in the output space, reducing uncertainty and enhancing generalization performance. Experiments on real-world datasets demonstrate the effectiveness of our approach.

## 1. Introduction

Recommendation systems is a useful tool to address information overload [1,2,3]. Multimedia recommendation systems (MRS) utilize user-item interactions and multimodal features such as text, images, audio, and videos to provide content recommendations based on user preferences [4,5,6,7]. They play a crucial role in platforms like e-commerce [8], social media [9], and video sharing [10], enhancing recommendation accuracy by capturing user preferences at the fine-grained level [4,11,12]. Early methods, such as VBPR [13] and DeepStyle [14], integrated multimodal information into traditional collaborative filtering paradigms but overlooked high-order user-item interaction connectivity [15]. Recent approaches, including MMGCN [16], GRCN [17], LATTICE [5], and DualGNN [18], employ graph convolution network (GCN) to better represent user-item interactions and improve recommendation performance [19,20].

**Figure 1:**
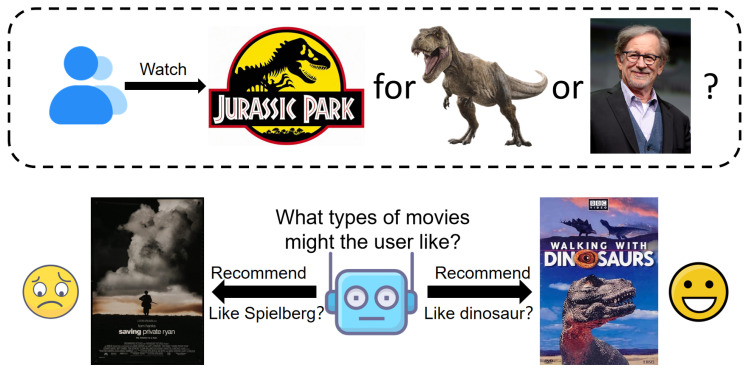
Schematic diagram of spurious correlation in MRS.

Despite progress, many multimedia recommendation methods face out-of-distribution (OOD) generalization issues, where models trained on one data distribution perform poorly when applied to data from a different distribution [21,22,23,24,25]. For instance, as shown in Figure 1, the user likes dinosaur movies, especially Jurassic Park, but if the movie is recommended based on the director, Spielberg, the user’s true preference for dinosaur themes is ignored. In this case, the association of Spielberg’s label with the user’s preferences is misleading, resulting in inaccurate recommendations. In other words, the Spielberg is the spurious texture feature, and the dinosaur is the causal texture feature.

To address these issues, invariant representation learning (IRL) has been proposed, aiming to learn features consistent across different environments [26], such as invariant risk minimization (IRM) [27,28,29]. However, in multimedia recommendation systems, methods like InvRL [30] and PaInvRL [31] may fail to fully align and interact between modalities, limiting recommendation performance.

Aligning modality-specific information is crucial for effective recommendations. However, simply aligning all modalities in a shared space is insufficient, as different modalities, such as audio, text, and images, capture various aspects of user preferences [32]. For example, in recommending an action movie, intense sound effects may indicate a preference for a tense atmosphere, text descriptions might reveal interest in the storyline, and posters or images could highlight an affinity for visual elements. Thus, integrating modality-specific information can prevent generalization issues associated with a single shared space. Another challenge associated with the alignment of modality-specific information is determining the contribution of each modality to the final prediction. To address this, a weighted fusion method is proposed, allowing the flexible adjustment of modality weights to ensure effective integration without over-reliance on any single modality.

In this paper, we propose the invariant representation learning in multimedia recommendation with modality alignment and model fusion framework ($M^{3}$-InvRL), which integrates multimodal representation, invariant learning, and model merging. Our approach introduces a novel contrastive representation learning method that decomposes each modality into common and specific components, extracting invariant features through environment identification and mask generation. These features are then merged and predicted for each modality, followed by weighted model merging in the output space. The main contributions of this paper are summarized as follows:

- We propose to learn both shared and modality-specific representations to mitigate the generalization issues of relying on a single shared space. By aligning individual modality representations with the complete set of modalities, the framework effectively integrates and complements information across modalities.
- We introduce a new multimedia recommendation framework, M^3^-InvRL, which maps modality features into shared and specific spaces to learn invariant representations for each component. We utilize model merging to fully leverage all available invariant information, adaptively adjusting the weights of different modality predictors to enhance the model’s generalization ability.
- We conduct extensive experiments on two real-world datasets to demonstrate the effectiveness of our proposed framework.

## 2. Related Work

### 2.1. Collaborative Filtering for Recommendation

Collaborative filtering (CF) is a foundational approach in recommendation systems, modeling the similarity between items and users to recommend similar items to similar users [33]. The core model in CF is matrix factorization (MF) [34], where each user and item is assigned a latent embedding, and similarity is assessed via the inner product of these embeddings. NCF [35] introduces neural networks to model similarities and proves that MF is a special case of NCF. NGCF [36] encodes high-hop neighbor information among users and items into embeddings using graph convolutional network (GCN). LightGCN [37] simplifies NGCF by removing feature transformations and nonlinear activations in the original NGCF architecture that are unsuitable for CF tasks. UltraGCN [38] further enhances efficiency by bypassing infinite layers of message passing in NGCF and LightGCN. We adopt UltraGCN as our backbone due to its simplicity and efficiency.

### 2.2. Multimedia Recommendation

Multimedia recommendation systems utilize multimodal information, such as visual, acoustic, and textual data, to enhance performance by better capturing user preferences [14,39,40]. Early works like VBPR integrated visual and item ID embeddings into a unified item embedding for further training [13]. DVBPR [41] extends the idea of VBPR by proposing an end-to-end architecture for jointly learning image representations and user-item embeddings. Later approaches introduced attention mechanisms to adaptively select multimodal features [42,43]. For instance, VECF [44] learns attention to sub-areas of images to make better image representations. UVCAN [45] uses attention mechanisms to learn multimodal information from both user and item perspectives. MAML [46] models each user’s attention to different aspects of an item by extracting multimodal features using an attention neural network. Recently, graph neural networks have been employed to model higher-order user-item interactions. MMGCN [16] learns modal-specific representations to better capture user preferences via the message-passing idea of GNN. LATTICE [5] constructs item–item graphs to improve item embeddings. However, these methods may fail when facing a distribution shift between training and test data, resulting in sub-optimal test performance.

### 2.3. Invariant Representation Learning

Invariant representation learning seeks to develop representations vital for downstream tasks, particularly by addressing distribution shifts between training and test data through consistent representations across diverse environments, thus improving generalization [47]. Invariant risk minimization (IRM) [27] is a seminal approach, with extensions in information theory [48,49], regularization [29,50], and sparsity [51]. Methods like EIIL [52] and HRM [53] automatically partition environments when labels are unavailable. Another approach involves constructing unbiased losses and optimizing models accordingly [54,55,56,57], including propensity score-based [58], doubly robust [59,60,61,62,63], and data fusion debiasing methods [64,65]. In this work, we capture invariant features using soft masks from heterogeneous environments and different modalities.

## 3. Preliminaries

In our multimedia recommendation model, the data mainly consists of two parts: users *u* and items *i*, which are represented by sets U and I, respectively. An interaction between a user and an item is represented as (u,i)∈U×I, where $r_{ui}$ represents the result of the interaction. If the interaction is positive, $r_{ui}=1$; otherwise, it is 0. The modal information for item *i* is represented as ${\left\{x_{M+1}^{i}=\left(x_{1}^{i},\cdots ,x_{M}^{i}\right)\right\}}_{i=1}^{N}$, where each $x_{m}^{i}\in R^{d_{m}}$ corresponds to a specific modality *m*. The parameter $d_{m}$ denotes the dimension of each modality. The multi-modal recommendation aims to learn a model $\Gamma \left(u,i,x_{M+1}^{i}|\Theta \right)$, where Θ denotes the parameters of the recommendation model Γ, to predict users’ true preferences.

(1) $$\begin{array}{c} \arg\min_{\Theta }L\left(\Gamma \left(u,i,x_{M+1}^{i}|\Theta \right)|R^{tr}\right), \end{array}$$

where L(·) denotes the recommendation loss, and $R^{tr}$ denotes the training set, with both positive samples $R^{+}=\left\{\left(u,i\right):r_{u,i}=1\right\}$ and negative samples $R^{-}=\left\{\left(u,i\right):r_{u,i}=0\right\}$. For easy reading, we provide the descriptions of all used variable in Table 1.

## 4. Methods

**Figure 2:**
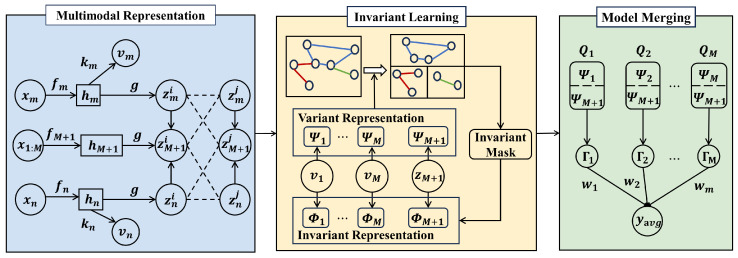
Overall framework of $M^{3}$-InvRL includes multimedia representation, invariant representation, and model merging.

In this section, we introduce the overall framework of M^3^-InvRL, as illustrated in Figure 2, which includes multimedia representation learning, invariant learning, and model merging.

### 4.1. Multimodal Representation for Recommendation

In this section, we first describe the modal-specific representation and one common representation for each modality. We introduce a novel contrastive loss that aligns the representation and imposes mutual information constraints to extract modality-specific features, preventing generalization issues within the same representation space. Next, we discuss the details of our method.

We use base encoders $f_{r}$ to generate *d*-dimensional representations $h_{r}=f_{r}\left(x_{r};\zeta_{r}\right)$ for r=1,…,M+1, where $h_{M+1}$ represents the intermediate representation of $x_{1:M}$. The shared head *g* maps these to a common space Z, generating shared representations $z_{m}=g\left(h_{m};\theta \right)$ for each modality and complete common representation $z_{M+1}=g\left(h_{M+1};\theta \right)$ for all modalities. Specific heads $k_{m}$ generate modality-specific representations $v_{m}=k_{m}\left(h_{m};\eta_{m}\right)$ for m=1,…,M.

We define sim(u,v) as the similarity measure between vectors *u* and *v*, such as cosine similarity $\mathrm{sim}\left(u,v\right)=\frac{u\cdot v}{∥u∥\;∥v∥}$. The similarity is scaled by a learnable temperature hyperparameter τ to yield the similarity score, where a larger τ reduces the distinction between similar and dissimilar samples, and a smaller τ enhances this difference. In our paper, τ helps balance the influence of positive and negative sample pairs.

(2) $$\begin{array}{c} s_{m,n}\left(i,j\right)=\exp\left(\mathrm{sim}\left(z_{m}^{i},z_{n}^{j}\right)/\tau \right), \end{array}$$

where $z_{m}^{i}$ and $z_{n}^{j}$ are the representations of the *m*th and *n*th modalities corresponding to the *i*th and *j*th samples from a mini-batch *B*, respectively.

We define $\left(z_{m}^{i},z_{M+1}^{i}\right)$ for i=1,⋯,B as positive pairs, the remaining pairs are the negative pairs,

(3) $$\begin{array}{cc} \Omega_{m}\left(i\right) & =\sum_{i\neq j}\left(s_{m,M+1}\left(i,j\right)+s_{m,m}\left(i,j\right)+s_{M+1,M+1}\left(i,j\right)\right) \end{array}$$

is the sum of similarities among negative pairs that correspond to the positive pair $\left(z_{m}^{i},z_{M+1}^{i}\right)$, and the contrastive loss for the same pair of samples is

(4) $$\begin{array}{c} l_{m}\left(i\right)=-\log\frac{s_{m,M+1}\left(i,i\right)}{\Omega_{m}\left(i\right)}. \end{array}$$

We combine the loss terms for each modality m=1,⋯,M and obtain the common loss

(5) $$\begin{array}{c} L_{\mathrm{com}}\left(B\right)=\sum_{m=1}^{M}\sum_{i=1}^{B}l_{m}\left(i\right). \end{array}$$

Aligning all modalities in a single shared space can lead to generalization issues and loss of unique modality-specific information. To preserve the distinctiveness of modality-specific representation $v_{m}\left(x\right)$ relative to modality-shared features $z_{m}\left(x\right)$, the goal is to minimize the mutual information

(6) $$L_{\mathrm{MI}}=\sum_{m=1}^{M}\mathrm{CLUB}\left(v_{m}\left(x\right),z_{m}\left(x\right)\right),$$

where CLUB(V,W) is the estimator for the contrastive log-ratio upper bound of mutual information between two random variables *V* and *W* [66].

### 4.2. Invariant Learning for Recommendation

Invariant learning [28,67] encourages models to concentrate on stable representations across different environments. Within our multimodal framework, it is applied to modality-specific representation $v_{m}$ and complete common representation $z_{M+1}$ to learn invariant representations ${\left\{\Phi_{r}\right\}}_{r=1}^{M+1}$.

**Environment Identification.** We take historical user-item interactions as input and partition them into a set of environments E, which supports the generation of invariant masks for the subsequent stages of learning.

During the environment identification stage, we aim to learn environment-specific representations $e_{r}\in E$ by training a recommendation model $\Gamma_{\left(e_{r}\right)}\left(u,i,\Psi_{r}^{i}|\Theta_{e_{r}}\right)$ for each environment $e_{r}$. Here, $\Psi_{r}^{i}$ denotes variant representations with item *i* and $\Theta_{e_{r}}$ represents the model’s parameters in the environment $e_{r}$:

(7) $$\begin{array}{c} \arg\min_{\Theta_{e_{r}}}L\left(\Gamma_{\left(e_{r}\right)}\left(u,i,\Psi_{r}^{i}|\Theta_{e_{r}}\right)|R_{e_{r}}^{tr}\right), \end{array}$$

where the variant representations $\Psi_{r}^{i}$ are obtained by initializing the invariant mask. We employ UltraGCN [38] as the recommendation model and drive the representations through a graph-based loss function L to encode the user-item graph.

Once the environment-specific representations are learned, the user-item interactions are assigned to the corresponding environments by maximizing the recommendation model output for each interaction:

(8) $$\begin{array}{c} R_{e_{r}}=\arg\max_{e_{r}\in E}\Gamma_{\left(e_{r}\right)}\left(u,i,\Psi_{r}^{i}|\Theta_{e_{r}}\right). \end{array}$$

The environment-specific interaction sets $\left\{R_{e_{r}}|e_{r}\in E\right\}$ are then used to guide the invariant representation learning.

**Invariant Representation Learning.** We minimize $L_{r}^{mask}$ by optimizing the mask m. To constrain that each $m_{i}$ in the mask is between [0,1], we use the softmax function.

Followed with the prediction model $\Gamma_{r}^{mask}$ converging, the invariant representations

(9) $$\begin{array}{cc} \Phi_{r}^{i} & =m_{r}^{i}⊙v_{r}^{i},r=1,\cdots ,M,\\ \Phi_{r}^{i} & =m_{r}^{i}⊙z_{r}^{i},r=M+1, \end{array}$$

and variant representations

(10) $$\begin{array}{cc} \Psi_{r}^{i} & =\left(1-m_{r}^{i}\right)⊙v_{r}^{i},r=1,\cdots ,M,\\ \Psi_{r}^{i} & =\left(1-m_{r}^{i}\right)⊙z_{r}^{i},r=M+1. \end{array}$$

### 4.3. Model Merging for Recommendation

In multimodal fusion, a key challenge is that the contribution of each modality to the final prediction is uncertain. To address this, we apply a weighted fusion strategy that adjusts the importance of each modality based on its uncertainty.

Our approach concatenates the invariant representation $\Phi_{M+1}$ in the common space and the model-specific invariant representation $\Phi_{m}$ to obtain the combined modality feature $Q_{m}$, defined as

(11) $$\begin{array}{c} Q_{m}=\left[\Phi_{m};\Phi_{M+1}\right]. \end{array}$$

Thus, we learn the final recommendation model $\Gamma_{m}^{*}\left(u,i,Q_{m}|\Theta_{m}^{*}\right)$ based on the combined representation $Q_{m}$ in each modality. The learning objective in Equation (1) can be rewritten as

(12) $$\begin{array}{c} \arg\min_{\Theta_{m}}L(\Gamma_{m}^{*}\left(u,i,Q_{m}|\Theta_{m}^{*}|R^{tr}\right). \end{array}$$

When one modality exhibits higher uncertainty in its predictions, it becomes more prone to making incorrect predictions. Consequently, we leverage the prediction uncertainty as a proxy to gauge the importance of each modality.

(13) $$\begin{array}{cc} \lambda_{m} & =-p_{m}^{T}\log p_{m}, \end{array}$$

where $p_{m}=\mathrm{softmax}\left(\Gamma_{m}^{*}\left(u,i,Q_{m}|\Theta_{m}^{*}\right)\right)$.

A higher entropy $\lambda_{m}$ indicates lower confidence in the prediction, leading to a smaller importance weight during the model merging process. Based on this, we calculate the importance weight for a *m*th modality predictor as

(14) $$\begin{array}{c} \omega_{m}=\frac{\exp\left(\max_{m=1,\cdots ,M}\lambda_{m}-\lambda_{m}\right)}{\sum_{i=1}^{M}\exp\left(\max_{m=1,\ldots ,M}\lambda_{m}-\lambda_{i}\right)}. \end{array}$$

The final prediction is obtained by aggregating the outputs of all predictors. We use a weighted sum to combine the predictions, ensuring that the weights sum to one. Specifically, the final result $Y_{\mathrm{avg}}$ is given by

(15) $$\begin{array}{c} Y_{\mathrm{avg}}=\sum_{m=1}^{M}\omega_{m}\Gamma_{m}^{*}\left(u,i,Q_{m}|\Theta_{m}^{*}\right). \end{array}$$

## 5. Results

### 5.1. Datasets

Following previous work [17,30,31,68], we conducted experiments using two publicly available multimedia datasets: **Tiktok** (https://github.com/nickwzk/InvRL, accessed on 10 October 2022) and **Movielens** (https://github.com/nickwzk/InvRL, accessed on 10 October 2022). The **Tiktok** dataset contains short micro-videos, while the **Movielens** dataset consists of user movie viewing histories. Both datasets include multimedia representations extracted from visual, acoustic, and textual content. The representations of the **Tiktok** dataset are extracted and provided officially. The visual, acoustic, and textual representations of the **Movielens** dataset were extracted by [16] with pre-trained ResNet50 for visual representations, VGGish [69] for acoustic representations, and [70] for textual representations. Note that there are many widely used datasets such as **Kwai** (https://github.com/nickwzk/InvRL, accessed on 10 October 2022) included in previous work. However, since such datasets only contain one modality, we excluded this dataset from our experiment. The summary statistics of the **Tiktok** and **Movielens** datasets are shown in Table 2.

### 5.2. Experiment Details

We adopted Adam [71] as the optimizer and implemented our models using PyTorch 1.11.0, running on an NVIDIA V100 GPU. The batch size was set to 512, and the number of environments was selected from {5, 10, 15, 20, 25}. The learning rate was tuned within the set {0.01, 0.001, 0.0001}. For the regularization parameters, $\lambda_{\mathrm{com}}$ was chosen from {0.1, 1, 2, 5, 10} and $\lambda_{\mathrm{MI}}$ from {0.01, 0.1, 1, 10}. Additionally, γ and ρ were selected from {0.01, 0.1, 0.5, 1, 5}, while κ and ν were chosen from {0.1, 1, 5}. The temperature hyperparameter τ was tuned within the range {0.1, 0.5, 1, 5, 10}. The iteration parameter *T* was initially set to 5, and training was conducted for 200 epochs.

### 5.3. Baselines

We evaluated our model against several state-of-the-art multimedia recommendation methods. The M-CF models, including VBPR [13], CB2CF [72], and DUIF [73], integrate multimedia content into traditional collaborative filtering approaches. G-NCF models, such as DisenGCN [74], MacridVAE [75], and NGCF [36], employ neural networks to capture complex user-item interactions. M-NCF models, including HUIGN [68], GRCN [17], and MMGCN [16], specialize in neural CF for multimedia content. InvRL models, such as InvRL [30], introduce invariant learning. UltraGCN [38] served as the backbone, simplifying graph CF through regularization and improving efficiency.

### 5.4. Evaluation Metrics

We used three widely-used evaluation metrics: Precision@K (P@K), Recall@K (R@K), and NDCG@K (N@K), to measure the ranking performance of our proposed method. Precision@K calculates the average of the proportion of the corrected recommended items among the top K predicted items for each user. Recall@K calculates the average of the proportion of the corrected recommended items among the sum of the corrected recommended items and the wrongly missed items in the top K predicted items for each user. NDCG@K, short for normalized discounted cumulative gain at K, measures the order of the corrected recommended items in the top K predicted items. Higher values of the three metrics indicate better ranking performance of our proposed method. In our experiments, K was set to 10.

### 5.5. Overall Performance

We report the performance of various methods on both **Tiktok** and **Movielens** datasets in Table 3, where the best-performing method is bolded for each metric. We have the following observations.

Firstly, multi-modality-based methods outperform single-modality-based methods, emphasizing the critical role of integrating multi-modality information to enhance recommendation performance. M^3^-InvRL achieves the most competitive performance among all the methods.

Secondly, compared to the Naive-UltraGCN, the incorporation of InvRL on UltraGCN (InvRL) enhances the recommendation performance through the introduction of invariant representation learning. On the other hand, our proposed M^3^-InvRL further enhances the recommendation performance on InvRL. On the **Movielens** dataset, M^3^-InvRL outperforms InvRL by 4.65% in Precision@10, 6.11% in Recall@10, and 0.89% in NDCG@10. On the **Tiktok** dataset, M^3^-InvRL surpasses InvRL with a 3.13% increase in Precision@10, 3.49% increase in Recall@10, and 3.79% increase in NDCG@10. We can conclude that unlike InvRL’s direct concatenation of representations, M^3^-InvRL achieves higher performance by aligning modalities through multimodal contrastive representation learning and applying model merging in each modality prediction model.

### 5.6. Performance Comparison with Different Values of *K*

**Figure 3:**
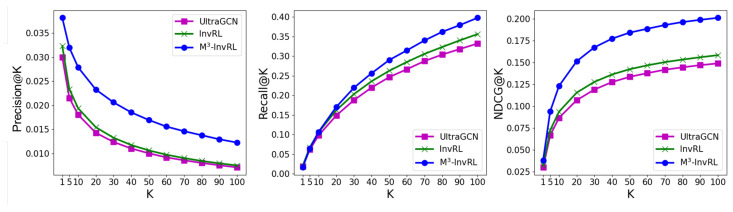
The comparison among Naive-UltraGCN (UltraGCN), UltraGCN + InvRL (InvRL) and $M^{3}$-InvRL on **Tiktok** datasets with respect to Precision@K, Recall@K, NCDG@K.

To highlight the improvements of $M^{3}$-InvRL, we conducted a comparative analysis between $M^{3}$-InvRL and its backbone model, UltraGCN, by evaluating their top-*K* scores. Figure 3 illustrates the curves for NDCG, prediction, and recall scores on the **TikTok** dataset.

$M^{3}$-InvRL consistently outperforms Naive-UltraGCN and UltraGCN + InvRL across all three metrics. Specifically, in Precision@K, $M^{3}$-InvRL demonstrates higher accuracy and maintains superior prediction scores across various K values, indicating its effectiveness in identifying the most relevant items at the top of the recommendation list. In Recall@K, $M^{3}$-InvRL achieves higher recall, particularly as K increases, showcasing its ability to retrieve more relevant items in scenarios where maximizing relevant item retrieval is essential. Finally, in NDCG@K, which considers both the relevance and ranking of recommended items, $M^{3}$-InvRL not only identifies relevant items but also ranks them more effectively, leading to significant performance improvements over other approaches. These consistent enhancements across different evaluation metrics underscore the robustness and effectiveness of $M^{3}$-InvRL in delivering high-quality recommendations.

### 5.7. Effect of $L_{\mathrm{com}}$ and $L_{\mathrm{MI}}$

In this section, we examine the impact of the common loss $L_{\mathrm{com}}$ and the mutual information loss $L_{\mathrm{MI}}$ on the model’s performance. We do this by removing each loss during the training process of $M^{3}$-InvRL. For comparative purposes, we evaluate the following three models: $M^{3}$-InvRL without the common loss $L_{\mathrm{com}}$ (denoted as $M^{3}$-InvRL w/o $L_{\mathrm{com}}$), $M^{3}$-InvRL without the mutual information loss $L_{\mathrm{MI}}$ (denoted as $M^{3}$-InvRL w/o $L_{\mathrm{MI}}$), and the original $M^{3}$-InvRL model. The experimental results are presented in Table 4.

Our observations indicate that removing the common loss $L_{\mathrm{com}}$ leads to a performance decline across both datasets. This highlights the crucial role of aligning common representations in multimodal representation learning. Similarly, the removal of the mutual information loss $L_{\mathrm{MI}}$ negatively affects the model’s performance. This suggests that relying on a single shared representation space may restrict the model’s generalization capabilities, underscoring the importance of $L_{\mathrm{MI}}$ in effectively capturing modality-specific features.

Furthermore, we note that the performance drop in $M^{3}$-InvRL w/o $L_{\mathrm{com}}$ is more pronounced than in $M^{3}$-InvRL w/o $L_{\mathrm{MI}}$. This demonstrates that common representations are pivotal in determining user preferences, while modality-specific representations play a significant supplementary role.

### 5.8. Different Model Merging Strategy

To validate the effectiveness of our proposed model merging strategy, we conducted experiments using three additional weighting methods: equal weighting (E-weight), loss-based weighting (L-weight), and attention mechanism-based weighting (A-weight). In the equal weighting strategy, each modality model is assigned an equal weight of 1/3. The loss-based weighting strategy builds upon this by assigning weights based on the ratio of each modality’s loss to the total loss across all modalities, thereby giving more importance to modalities that contribute less error. The attention mechanism-based weighting further enhances the approach by dynamically adjusting weights according to the relevance of each modality’s information.

As shown in Table 5, the loss-based strategy performs almost identically to equal weighting, indicating that merely acknowledging the differences between modalities does not improve overall performance. However, the attention mechanism strategy significantly enhances model performance compared to both the loss-based and equal weighting strategies. This suggests that by dynamically adjusting weights based on the importance of each modality in varying contexts, the merging mechanism can improve model performance. Furthermore, the $M^{3}$-InvRL model achieves the best performance across all metrics. By employing entropy-based weights as a proxy for model uncertainty, $M^{3}$-InvRL dynamically and accurately allocates weights, effectively leveraging the strengths of each modality and reducing uncertainty. This leads to superior overall performance, as demonstrated by the experimental results.

### 5.9. Study on the Number of Environments

**Figure 4:**
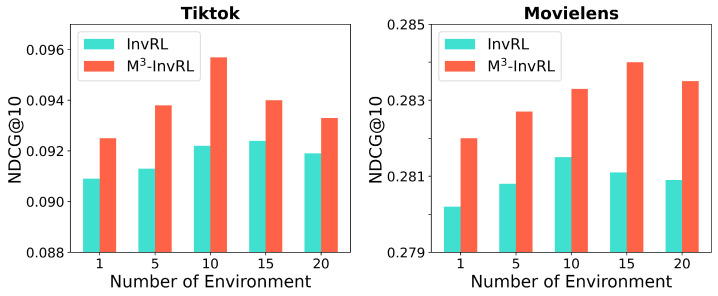
Experimental comparison of different environment numbers |E|.

To assess how the number of environments impacts the performance of $M^{3}$-InvRL compared to InvRL, we conducted experiments on the **Tiktok** and **Movielens** datasets with varying numbers of environments. As illustrated in Figure 4, $M^{3}$-InvRL consistently surpasses InvRL in NDCG@10 across different environment counts. A key advantage of $M^{3}$-InvRL is its use of weighted averaging after adapting to each modality’s environment, which reduces the model uncertainty and enhances flexibility. In contrast, InvRL simply concatenates modes as a representation to learn invariant representations. By learning invariant representations separately from specific and common complete representations, $M^{3}$-InvRL facilitates easier differentiation between environments. In the **Tiktok** dataset, using approximately 10 environments yields the best performance, as this number allows $M^{3}$-InvRL to effectively distinguish between variant and invariant information, thereby enhancing recommendation quality. For the **Movielens** dataset, the performance improves with an increasing number of environments, suggesting that a larger number of environments is more suitable for this dataset.

## 6. Discussion

In this work, we propose an invariant representation learning framework ($M^{3}$-InvRL) to enhance the generalization ability of multimedia recommendation systems, particularly in the presence of distribution shifts between training and testing data. Our method achieves up to an 8.95% improvement in ranking performance on the **Movielens** dataset and a 12.03% improvement on the **Tiktok** dataset over the Naive-UltraGCN model. Compared to the UltraGCN + InvRL method, our approach yields up to a 6.11% improvement on the **Movielens** dataset and 3.79% on the **Tiktok** dataset. These improvements stem from three key components of our framework.

The first component involves the separation of common and modality-specific representations. For each modality, we use different heads to transform the original representation into common and modality-specific parts. A common loss and a mutual information loss are then combined to enhance the representation capabilities of the common representation and the distinctiveness of the modality-specific representation relative to the shared representation. This separation guides the model to learn more representative features for downstream tasks. Experiments demonstrate that both types of representations contribute to the model’s performance. The second component is invariant representation learning applied to both common and modality-specific representations. This approach endows our model with the ability to maintain robustness when faced with distribution shifts between training and testing data. The third component involves model merging through an ensemble of modality-level predictions. Unlike existing works [30] that train a single model on concatenated features from multiple modalities, we train a distinct model for each modality to capture modality-specific information and merge the results based on their importance to overall performance. This enables our model to learn and adjust its focus on relevant information.

Despite the advantages of our proposed method, there are still improvements that can be made in the future. The first one is the determination of the number of environments. In this work, we predefined the number of environments, but optimal numbers vary across datasets. Developing an adaptive method to automatically determine the number of environments would be beneficial. Second, we may enhance the efficiency of our method, as dividing it into three consecutive parts may increase the training costs. An end-to-end approach that integrates these components could significantly improve the efficiency in the future.

## 7. Conclusions

Our $M^{3}$-InvRL framework enhances the generalization ability of multimedia recommendation systems in the presence of distribution shifts between training and testing data. Specifically, our approach learns both shared and modality-specific invariant representations. By utilizing modal-specific and common representations, invariant learning, and adaptive model merging techniques, our method effectively addresses issues related to spurious feature learning and misalignment.

## Figures and Tables

**Table 1:** List of all variables used in this paper and their corresponding descriptions.

Variable	Description
*u*, U	User *u* in the recommendation system, and U is the set of all users.
*i*, I	Item *i* in the recommendation system, and I is the set of all items.
$r_{ui}$	Binary interaction: $r_{ui}=1$ if user *u* positively interacts with item *i*, 0 otherwise.
R	Set of all user-item interactions, where $R^{+}$ denotes positive samples ($r_{ui}=1$), and $R^{-}$ denotes negative samples ($r_{ui}=0$).
$x_{m}^{i}$	Feature of item *i* for modality *m*, m∈{1,⋯,M}.
$d_{m}$	Dimension of the feature vector for modality *m*.
Γ(·)	Recommendation model predicting user preferences.
Θ	Parameters of the recommendation model Γ.
$f_{r}\left(\cdot \right)$	Base encoder for the *r*-th modality, r=1,⋯,M+1.
$h_{r}$	Representation generated by $f_{r}\left(\cdot \right)$ for the *r*-th modality.
g(·)	Shared head mapping representations to a common space Z.
$z_{m}$, $z_{M+1}$	Shared representations for modality *m* and all modalities, respectively.
$k_{m}\left(\cdot \right)$	Specific head generating modality-specific representations.
$v_{m}$	Modality-specific representation for modality *m*.
$s_{m,n}\left(i,j\right)$	Similarity score between modality *m* (sample *i*) and modality *n* (sample *j*).
$L_{\mathrm{com}}\left(B\right)$, $L_{\mathrm{MI}}$	Common loss across modalities and mutual information loss.
$\Phi_{r}^{i}$, $\Psi_{r}^{i}$	Invariant and variant representations for modality *r* of item *i*.
m	Invariant mask in [0,1], used to generate invariant representations.
$Q_{m}$	Combined invariant representations for modality *m*, $Q_{m}=\left[\Phi_{m};\Phi_{M+1}\right]$.
$\Gamma_{m}^{*}\left(\cdot \right)$	Final recommendation model for modality *m*, trained on $Q_{m}$.
$\lambda_{m}$	Entropy-based uncertainty for the *m*-th modality.
$\omega_{m}$	Importance weight for the *m*-th modality.
$Y_{\mathrm{avg}}$	Final prediction by aggregating all predictors.

**Table 2:** The statistics of datasets. $d_{V}$, $d_{A}$, and $d_{T}$ denote the dimensions of visual, acoustic, and textual modalities. # means “the numbers of”.

Dataset	#Interactions	#Items	#Users	Sparsity	$d_{V}$	$d_{A}$	$d_{T}$
**Movielens**	1,239,508	5986	55,485	99.63%	2048	128	100
**Tiktok**	726,065	76,085	36,656	99.99%	128	128	128

**Table 3:** Performance comparison across datasets using Precision@10, Recall@10, and NDCG@10. The best result is bold. The second best result is underlined.

Category	Methods	Movielens			Tiktok		
		**P@10**	**R@10**	**N@10**	**P@10**	**R@10**	**N@10**
**M-CF**	VBPR	0.0512	0.1990	0.2261	0.0118	0.0628	0.0574
	DUIF	0.0538	0.2167	0.2341	0.0087	0.0483	0.0434
	CB2CF	0.0548	0.2265	0.2505	0.0109	0.0642	0.0613
**G-NCF**	NGCF	0.0547	0.2196	0.2342	0.0135	0.0780	0.0661
	DisenGCN	0.0555	0.2222	0.2401	0.0145	0.0760	0.0639
	MacridVAE	0.0576	0.2286	0.2437	0.0152	0.0813	0.0686
**M-NCF**	MMGCN	0.0581	0.2345	0.2517	0.0144	0.0808	0.0674
	HUIGN	0.0619	0.2522	0.2677	0.0164	0.0884	0.0769
	GRCN	0.0639	0.2569	0.2754	0.0195	0.1048	0.0938
**UltraGCN**	Naive-UltraGCN	0.0624	0.2547	0.2691	0.0183	0.0981	0.0878
	UltraGCN + InvRL	0.0645	0.2615	0.2815	0.0192	0.1062	0.0922
	$M^{3}$ **-InvRL(Ours)**	**0.0675**	**0.2775**	**0.2840**	**0.0198**	**0.1099**	**0.0957**
%Improvement over Naive-UltraGCN		8.17%	8.95%	5.55%	8.20%	12.03%	9.00%
%Improvement over UltraGCN + InvRL		4.65%	6.11%	0.89%	3.13%	3.49%	3.79%

**Table 4:** Performance comparison with different loss components. The best result is bold. The second best result is underlined.

	Movielens			Tiktok		
	**P@10**	**R@10**	**N@10**	**P@10**	**R@10**	**N@10**
M^3^-InvRL w/o $L_{\mathrm{com}}$	0.0642	0.2648	0.2792	0.0190	0.1030	0.0925
M^3^-InvRL w/o $L_{\mathrm{MI}}$	0.0667	0.2753	0.2836	0.0194	0.1093	0.0931
M^3^-InvRL	**0.0675**	**0.2775**	**0.2840**	**0.0198**	**0.1099**	**0.0957**

**Table 5:** Performance comparison on different weight strategies. The best result is bold. The second best result is underlined.

	Movielens			Tiktok		
	**P@10**	**R@10**	**N@10**	**P@10**	**R@10**	**N@10**
E-weight	0.0652	0.2731	0.2829	0.0192	0.1080	0.0911
L-weight	0.0648	0.2719	0.2817	0.0193	0.1073	0.0937
A-weight	0.0670	0.2761	0.2834	0.0195	**0.1105**	0.0955
M^3^-InvRL	**0.0675**	**0.2775**	**0.2840**	**0.0198**	0.1099	**0.0957**

## Data Availability

Restrictions apply to the availability of these data. Data were obtained from GitHub and are available https://github.com/nickwzk/InvRL (accessed on 10 October 2022) with the permission of GitHub.
